# Editorial: Applications of biomimetic (composite) materials

**DOI:** 10.3389/fbioe.2022.1020909

**Published:** 2022-09-13

**Authors:** Yi-Chen Ethan Li, Jeng-Shiung Jan, PIotr Lulinski, Hung-Yin Lin

**Affiliations:** ^1^ Department of Chemical Engineering, Feng Chia University, Taichung, Taiwan; ^2^ Department of Chemical Engineering, National Cheng Kung University, Tainan, Taiwan; ^3^ Department of Organic Chemistry Faculty of Pharmacy, Medical University of Warsaw, Warsaw, Poland; ^4^ Department of Chemical and Materials Engineering, National University of Kaohsiung, Kaohsiung, Taiwan

**Keywords:** biomimetic composite materials, tissue engineering, biomimetic robot, regenerative medicine, drug deliver system

This special issue summarizes the current trends in the development of biomimetic composite materials for tissue engineering and regenerative medicine applications. In a typical biomaterials approach, two-dimensional (2D) or three-dimensional (3D) culture systems made from different types of biomaterials have attracted more and more interest from tissue engineers (Ma 2008). Through controlling the mechanical, porosity, and spatial structures of biomaterials, rapid development in 2D and 3D biomimetic environments advance the creation of natural biomimetic systems for use in the field of drug delivery, targeted therapy, tissue engineering, bioimaging, and sensing because of the continuous need to improve the quality of life, to provide personalized treatment, to find lesions as early as possible and to detect biomarkers at trace levels.

Biomimetic materials are synthetic or modified natural materials that mimic natural materials or follow a design motif derived from nature and are also very useful in designing composite materials to solve human problems (Chen et al., 2022). Moreover, nature offers many examples to guide engineers to simultaneously achieve higher levels of structural and functional properties. However, the development of bioinspired natural materials requires a combination of different properties of materials, which is insufficient to generate high-performance materials and achieve the desired functions through modifying the properties of single material (Sang et al., 2018). It implies the need to develop composite materials to fulfill human requirements and improve the quality of human life.

Bittencourt et al. gave an overview of the relationships between structural properties and function of spider silks for designing and generating spidroins through the principle of synthetic biology. Furthermore, they discussed the molecular structure effect of spidroins on the mechanical properties of materials, offering a summary of the recent progress in producing innovative structural materials by using recombinant bioengineered spidroins.

Li and co-workers designed mussel-inspired nanocomposite polydopamine-based materials with good conductivity, high fluorescence quantum yields, and low cytotoxicity to regulate cell behaviors. Furthermore, they demonstrated that the conductive mussel-inspired nanocomposite materials have a promising potential to set an electroactive platform to enhance cell adhesion and growth and remove harmful cells via electronic control procedures.

Yao and co-workers combined a tricalcium phosphate (TCP)-based gelatin scaffold (GGT) and electroacupuncture stimulation to accelerate the bone remodeling process. They focused on investigating the dual effect of chemical and physical stimuli from the composite scaffold and electrical stimulation on new bone regrowth *in vitro* and *in vivo*. The study provides potential interest in combining composite biomaterials and external stimulation to improve bone defects after injury.

Xu et al. indicated the importance of experimental models precisely recapturing liver functions and reflecting the hepatotoxicity effect of the drug on the liver and the necessity of a 3D biomimetic composite material-based system for evaluating liver hepatotoxicity. Specifically, their composite hydrogel fibers provided a method to fabricate a 3D hepatic plate-like co-culture system that can recapture the characters of hepatic plate-like structure and have a bile secretion function applicable for chronic/acute drug hepatotoxicity evaluation.

Borihindakul and his co-workers reviewed the features of two types of foot microstructures, spatula-shaped and mushroom-shaped, capable of generating adhesive foot. Subsequently, they discussed the technologies of dry, wet, magnetic, and pneumatic adhesion and the applications of bio-inspired adhesion in the design of climbing robots. Furthermore, they described the relationship between spatula- and mushroom-shaped microstructures and the creation of climbing robots that guide roboticists in selecting the adequate adhesive foot to reach the desired climbing ability for robot developments in the future.

Khan et al. developed a bioinspired hybrid rigid-soft robot-foot from the morphology of inchworm legs to overcome the issue that few legged robots can crawl on curved metal pipe surfaces. They described that the robot foot consists of a rigid electromagnet part, and a soft toe covering endows the robot with the ability to be a metal pipe crawler. The study provides a strategy to design the robots meeting the requirements of crawling large oil and gas pipelines in inspecting the leakages and faults.

Zhang et al. reviewed a topic about the role of the gecko tail in helping adaptive and robust climbing behaviors on sloped walls. Furthermore, they addressed the role of gecko and robot tails in the climbing performances. Moreover, they also analyzed and compared the feasibility of rigid and soft composite materials for designing the robots’ tails. The article provided the information to design and fabricate the climbing robots and addressed whether the tails are required for robots and the concept of selecting the adequate materials for the tails according to the motion types and desired functions in different environments.

In conclusion, we would like to thank the participants for their contributions. We hope that this special issue will be helpful for the development of novel biomimetic composite materials in biomedical applications.
